# Lack of association between the −2549 insertion/deletion variant of vascular endothelial growth factor and coronary artery disease in the Turkish population

**DOI:** 10.1590/1806-9282.20240333

**Published:** 2024-11-11

**Authors:** Serbulent Yigit, Ayse Feyda Nursal, Atac Celik, Recai Aci, Elgiz Askeroglu

**Affiliations:** 1 Ondokuz Mayıs University, Faculty of Veterinary Medicine, Department of Genetics – Samsun, Turkey.; 2 Tokat Gaziosmanpaşa University, Faculty of Medicine, Department of Internal Medicine, Department of Medical Genetics – Tokat, Turkey.; 3 Tokat Gaziosmanpasa University, Faculty of Medicine, Department of Cardiology – Tokat, Turkey.; 4 Adnan Menderes University, Söke Vocational School of Health Services – Aydın, Turkey.; 5 Giresun University, Faculty of Arts and Sciences, Department of Statistics – Giresun, Turkey.

**Keywords:** Coronary artery disease, Vascular endothelial growth factor, Variant, PCR

## Abstract

**OBJECTIVE:**

Coronary artery disease is the leading cause of death worldwide. Vascular endothelial growth factor is known to induce endothelial cell migration and proliferation, increase vascular permeability, and modulate thrombogenicity. The aim of this study is to investigate the relationship between the *VEGF* insertion/deletion (I/D) variant (rs35569394) and coronary artery disease susceptibility in the Turkish population.

**METHODS:**

A total of 206 subjects, including 106 coronary artery disease patients and 100 controls, were included in this study. The *VEGF* I/D variant was genotyped using the polymerase chain reaction method.

**RESULTS:**

The frequency of the I/I, I/D, and D/D genotypes was 35.84 versus 37%, 33.97 versus 36%, and 30.19 versus 27% in patients and the control group, respectively. *VEGF* I/D genotype and allele distribution were not statistically significant between coronary artery disease patients and controls (p>0.05). There was no significant difference between *VEGF* I/D genotype distribution and patient characteristics including age, gender, disease duration, *high-density lipoprotein* cholesterol, *low-density lipoprotein* cholesterol, triglyceride, history of hypertension, history of diabetes mellitus, and smoking (p>0.05).

**CONCLUSION:**

This study suggests that the *VEGF* I/D variant is not a predisposing factor to coronary artery disease disease in a Turkish sample.

## INTRODUCTION

Coronary artery disease (CAD) is a major cause of global morbidity and mortality. In this disease, the coronary arteries that supply blood and oxygen to the heart become narrowed or blocked due to a thrombus or spasm^1^. Atherosclerosis is characterized by plaque accumulation in the vascular endothelium and is an important cause of CAD^2^. Atherosclerosis results from complex interactions between risk factors such as hypertension (HTN), tobacco use, dyslipidemia, and diabetes. With chronic exposure to risk factors, the defense mechanisms of the vascular endothelium are weakened, and its integrity is impaired. As a result, endothelial cell (EC) dysfunction occurs, which is important in the pathogenesis and complications of atherosclerosis^3^. 40–60% of CAD susceptibility is thought to reflect genetic risk^4^. In genome-wide association studies (GWASs), some genes associated with blood pressure, lipid metabolism, obesity, insulin resistance, neovascularization and angiogenesis, immunological response and inflammation, thrombosis, and vascular remodeling have been found to be associated with CAD^5^. One of the genes associated with neovascularization and angiogenesis is vascular endothelial growth factor (VEGF)^6^.

VEGF-A, VEGF-B, and placental growth factors, which are important blood vessel growth regulators, are members of the human VEGF family. Vasculogenesis and angiogenesis vessels are mostly dependent on VEGF-A (also called VEGF)^7^. VEGF probably has a protective function in CAD because angiogenesis can act as a compensatory mechanism to reduce myocardial ischemia during severe coronary atherosclerotic diseases^8^. The *VEGF* gene is located at chromosome 6p21.3 and has a 14-kb coding sequence with seven introns and eight exons. Due to its high polymorphism, it has many functional single-nucleotide polymorphisms (SNPs) in the promoter regions, 3'-untranslated region (UTR), and 5'-UTR^9^. It has been shown in vivo that functional variants in VEGF may play an important role in coronary artery atherosclerosis by altering VEGF levels. The insertion/deletion (I/D) polymorphism of the 18-bp fragment at position −2549 of the *VEGF* gene promoter region (rs35569394) has been shown to predispose to many angiogenic-based diseases^10^.

Therefore, we aimed to investigate whether the *VEGF* I/D variant differs between patients with CAD and controls in the Turks.

## METHODS

### Subjects

A total of 160 patients with symptomatic CAD (81 males and 25 females; mean age: 62.12±9.15 years) from the Department of Cardiology, Gaziosmanpasa Medical Faculty, Tokat, Turkey. Only individuals with myocardial infarction (MI) symptoms, angina, a history of previous angioplasty, CAD bypass grafting, or more than 30% stenosis in one or more coronary arteries were included in this study. Nevertheless, the study did not include participants with cardiomyopathy, valvular heart disease, congenital heart disease, or stroke. Clinical information of the patients, including *high-density lipoprotein (*HDL) cholesterol, *low-density lipoprotein (*LDL) cholesterol, smoking, HTN, diabetes mellitus, triglyceride, and disease duration, was obtained. Moreover, 100 individuals (64 males and 36 females; mean age: 58.2±9.85 years) without a history of cardiovascular disease (CVD) comprised the control group. Participants who matched in terms of age and gender were chosen. Every study participant provided informed consent before donating a blood sample for genetic analysis. The Declaration of Helsinki and its subsequent revisions’ ethical guidelines were followed in the execution of the study since the protocol was accepted by the local ethics committee.

### Genotyping

**Figure 1:**
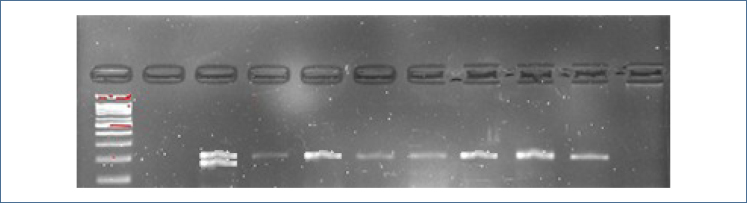
Polymerase chain reaction gel image.

Each participant, both patients and controls, provided 2 mL of venous blood for analysis. DNA extraction was conducted using a commercial kit following the manufacturer's instructions. The polymerase chain reaction (PCR) method was employed to identify the VEGF I/D variation using genomic DNA, as detailed previously (Figure 1)^10^. Visualization of the amplified products was performed using a 2.5% agarose gel. The amplified PCR products of VEGF resulted in 229 bp fragments representing the D allele and 211 bp fragments representing the I allele. To ensure the accuracy of the results, 20% of randomly selected samples were reanalyzed, and a 100% match was observed, validating the consistency of the findings.

### Statistical analysis

Statistical analysis was conducted utilizing the Statistical Package Program for the Social Sciences (SPSS, version 22). The results are presented as the mean±standard deviation (SD). The association between this variant and the clinical and demographic characteristics of patients was examined using the χ^2^ test, Mann-Whitney U test, Kruskal-Wallis, or analysis of variance statistics. The odds ratio and 95% confidence interval were employed to assess risk factors. All p-values were two-tailed, and significance was defined as p=0.05.

## RESULTS

In the present study, a total of 206 subjects, including 106 CAD patients and 100 controls, were genotyped for the *VEGF* I/D variant. The baseline demographic features of the subjects are shown in Table 1. Age was higher in the patient group than in the control group (p=0.007), while LDL levels were lower in the patient group compared to healthy controls (p=0.001). The history of HTN in the patients was higher than compared to that of the controls (p=0.0001).

**Table 1:** Baseline clinical and demographic characteristics of the patients with coronary artery disease and controls.

Characteristics	Patient group n=106 (%)	Control group n=100 (%)	p-value
Gender, male/female, n (%)	81/25 (76.4/23.6)	64/36 (64/36)	0.051
Age (years), mean, SD	62.12±9.15	58.2±9.85	**0.007**
HDL cholesterol, mean, SD (mg/dL)	45.07±12.48	45.08±11.51	0.655
LDL cholesterol, mean, SD (mg/dL)	114.42±38.56	132.67±38.30	**0.001**
Ever smoking, n (%)	18 (17)	28 (28)	0.058
History of hypertension, n (%)	77 (72.6)	49 (49)	**0.0001**
History of diabetes mellitus, n (%)	30 (28.3)	24 (24)	0.492

Statistically significant values are shown in bold. SD: standard deviation; HDL: high-density lipoprotein; LDL: low-density lipoprotein.

Allelic and genotypic distributions of the *VEGF I/D* variant in patients and controls are shown in Table 2. The frequency of the I/I, I/D, and D/D genotypes was 35.84 versus 37%, 33.97 versus 36%, and 30.19 versus 27%, in patients and the control group, respectively. There was no statistically significant difference between CAD patients and control subjects in the genotype and allele distribution of the *VEGF* I/D variant (p=0.877 and p=0.659, respectively). Also, no statistically significant difference was found between the patients and the controls in terms of the D/D+I/D genotype vs. I/I genotype and I/D+I/I vs. D/D genotype (p=0.864 and p=0.613, respectively).

**Table 2:** Genotype and allele distribution of vascular endothelial growth factor I/D in groups.

VEGF I/D		Patient group n=106 (%)	Control group n=100 (%)	p-value	OR (95%CI)
Genotypes					
	I/I	38 (35.84)	37 (37)		
	I/D	36 (33.97)	36 (36)		
	D/D	32 (30.19)	27 (27)		
	D/D+I/D: I/I	68 (67.4):38 (38.6)	63 (63.6):37 (36.4)	0.864	0.95 (0.54–1.68)
	D/D: I/D+I/I	32 (30.2):74 (69.8)	27 (27):73 (73)	0.613	0.85 (0.47–1.57)
Alleles					
	D	100 (47.17)	90 (45)	0.659	0.92 (0.62–1.35)
	I	112 (52.83)	110 (55)		

VEGF: vascular endothelial growth factor.

The study examined the relationship between the clinical and demographic characteristics of patients with CAD and the genotype distribution of the VEGF I/D gene. The analysis revealed no statistically significant differences between the VEGF I/D genotype distribution and various patient factors, such as age, gender, duration of the disease, levels of HDL cholesterol, LDL cholesterol, and triglycerides, as well as the history of HTN, diabetes mellitus, and smoking (p>0.05). The specific results are presented in Table 3.

**Table 3:** Clinical and demographic characteristics of coronary artery disease patients according to the vascular endothelial growth factor I/D genotype distribution.

VEGF I/D						
Characteristics		Total n=106 (%)	D/D n=32 (%)	I/D n=36 (%)	I/I n=38	p-value
Gender, male/female		81/25 (76.4/23.6)	25/7 (78.1/21.9)	30/6 (80.3/16.7)	26/12 (68.4/31.6)	0.308
Age (years)		62.12±9.15	63.96±9.48	61.05±7.75	61.57±10.06	0.386
Disease duration (years)		4.65±3.15	4.34±3.22	4.38±2.83	5.15±3.38	0.476
HDL cholesterol, mean, SD (mg/dL)		45.07±12.48	44.21±10.53	44.02±13.62	46.78±13,00	0.425
LDL cholesterol, mean, SD (mg/dL)		114.42±38.56	107.15±33.54	124.02±46.08	111.44±33.52	0.166
Triglyceride (mg/dL)		163.96±90,62	153.46±83.73	159.88±70.70	176.65±111.48	0.671
History of hypertension						
	Yes	77 (72.6)	26 (81.3)	21 (58.3)	30 (78.9)	0.059
	No	29 (27.4)	6 (18.8)	15 (41.7)	8 (21.1)	
History of diabetes mellitus						
	Yes	30 (28.3)	8 (25)	11 (30.6)	11 (28.9)	0.874
	No	76 (71.1)	24 (75)	25 (69.4)	27 (71.1)	
Smoking						
	Yes	18 (17)	6 (18.8)	8 (22.2)	4 (10.5)	0.388
	No	88 (83)	26 (81.3)	28 (77.8)	34 (89.5)	

VEGF: vascular endothelial growth factor; HDL: high-density lipoprotein; SD: standard deviation; LDL: low-density lipoprotein.

## DISCUSSION

In recent years, there has been an increase in deaths due to noncommunicable diseases globally. European Cardiovascular Statistics show that CVD is the leading cause of death in Europe, especially in middle-income countries^11^. Turkey is a developing Eurasian country with a population of 85 million in the Eastern Mediterranean region. According to 2022 data, ischemic heart disease accounted for 42.3% of deaths caused by circulatory system diseases in Turkey^12^. Numerous clinical and epidemiological investigations have shown the significance of genetic variables in the etiology of CAD. Based on family-based association studies, twin studies, or GWASs, the estimated heritability of CAD is 40–60%^13^. As a result of GWAS, it has been shown that there are many SNPs associated with CAD^13^. Sitinjak et al. reported that information on SNPs on CAD risk factors can be used as biomarkers for diagnosis and prediction of therapeutic responses to determine successful treatment and as the basis for defining personalized medicine in the future^14^.

VEGF mediates vascular permeability, angiogenesis, and inflammation through activating VEGFR-1 and VEGFR-2. The ECs, angioblasts, and pericytes in the cardiovascular system are the main locations where VEGF is expressed^15^. VEGF has a positive effect on revascularization through mechanisms such as the selective mitogenic effect on ECs, stimulation of the expression of vascular ECs, proliferation, regeneration, increase of vascular permeability, vasodilatation by activating nitric oxide (NO) synthase and prostacyclin, and inhibition of apoptosis. The antithrombotic properties of VEGF are revealed by the activation of serine proteases, urokinases, plasminogen activators, and the production of thrombolytic enzymes^16^. In most clinical studies, VEGF levels have been found to be increased in acute myocardial infarction compared to healthy individuals and patients with stable or unstable angina^17,18^. In CVDs, cardiomyocytes and ECs are frequently subjected to low oxygen levels (hypoxia) and inflammation. These conditions activate hypoxia-inducible factors (HIFs), which can upregulate various pro-angiogenic (blood vessel-promoting) factors, especially VEGF, through the HIF-1α pathway. Increased VEGF-A stimulates the proliferation of vascular ECs, enhances vascular permeability, and restores the integrity and function of the endothelium. This helps to compensate for the ischemia (restricted blood flow) and hypoxia and protects the damaged heart muscle^19^.

The latest meta-analysis on SNPs in the human VEGF-A gene found that the rs699947, rs1570360, and rs3025039 variants were linked to an increased risk of CAD. Additionally, the rs699947 and rs2010963 SNPs were identified as potential biomarkers for predicting poor collateral coronary circulation development following myocardial ischemia^20^. The genetic variant VEGF-A rs2010963 was found to be associated with an increased likelihood of developing MI^21^. The meta-analysis examined the connection between heart failure (HF) and SNPs in the VEGF gene. The findings indicated that the SNP in the rs748431 region of the FGD5 gene, which encodes a regulator of VEGF, was associated with an increased risk of hospital readmission and mortality in individuals with HF^22^.

The human *VEGF* gene is highly polymorphic, and some polymorphisms can affect expression between individuals^23^. There are many studies investigating the relationship between *VEGF* gene polymorphisms and CAD risk. A study conducted by Wang et al. found that two *VEGF* SNPs (rs699947 and rs3025039) were not associated with CAD in the Chinese population^24^. However, Han showed that *VEGF* −460T/C and −634G/C polymorphisms were significantly associated with CAD risk in another Chinese population^25^. A pooled analysis of a meta-analysis showed that VEGF rs699947, rs2010963, and rs3025039 were associated with an increased risk of CAD, whereas no significant association was observed with rs1570360^26^. Another meta-analysis showed a significant association between *VEGF* rs699947 and an increased risk of CAD^19^. A significant association was found between VEGF rs3025039 and CAD risk after stratifying by ethnicity and CAD type. The *VEGF* rs2010963 G/C polymorphism was also associated with MI risk. Li et al. demonstrated that *VEGF* rs699947 is related to CAD pathogenesis^27^.

The *VEGF* I/D has been shown to play a role in many angiogenic-based diseases and has therefore become the focus of attention. In this variant, which affects the gene expression, the del allele leads to a 1.95-fold increase in transcriptional activity compared to the ins allele^10^. Some studies have shown that people with D/D and I/D genotypes have higher serum VEGF levels^28,29^. In this study, we evaluated whether *VEGF* I/D predisposes to CAD in a Turkish sample. We found that the *VEGF* I/D genotype and allele distribution were not associated with CAD. Then we evaluate the relationship between genotype distribution and clinical findings. However, no relationship was found between *VEGF* I/D genotype distribution and patient demographic and clinical characteristics.

Our study has some limitations. We acknowledge that the small sample size is one of the limitations of the study. Missing data on some parameters may have prevented us from understanding their relationship with genotype. Since the study group consisted of Turkish patients, other populations cannot be predicted with certainty. Additionally, the medications used by the patients were not questioned.

## CONCLUSION

The development of CAD is attributed to the complex interaction of genetic and environmental factors. Our results showed that the *VEGF* I/D variant was not associated with the development of CAD.
